# Autism: a (key) piece of the global mental health puzzle

**DOI:** 10.1017/gmh.2014.7

**Published:** 2015-03-12

**Authors:** M.J. Rosanoff, A.M. Daniels, A. Shih

**Affiliations:** Autism Speaks, 1 E 33rd Street, 4th Floor, New York, NY, USA

**Keywords:** Autism Spectrum Disorder, Developmental Disabilities, Global Health, Mental Health, Public Health, Health Services, Capacity Building, Parent Training, World Health Organization, Autism Speaks

Once considered rare, autism is today recognized as an emerging global public health issue. Though the term emerging is traditionally reserved for describing new or rapidly spreading infectious diseases, the recent upsurge in reported prevalence has communities and public health officials worldwide scrambling to uncover ‘cause(s)’ of the autism ‘epidemic.’ Recent research suggests that broadening diagnostic criteria and changes in reporting practice can explain a portion, but not all, of the increase in prevalence over time (Hansen *et al.* 2015). Regardless of whether autism rates are truly rising and why, there has been an undeniable increase in global awareness as a consequence of these unanswered questions. You can hardly go a day without seeing a story on autism appear in mainstream media. And you would be hard pressed to find a major landmark not lit up blue for World Autism Awareness Day on April 2nd. Some, however, wonder if autism is receiving a disproportionate amount of attention. After all, it is just one in a host of childhood developmental and mental health conditions impacting communities in every corner of the world, every day. We believe that there is a tremendous opportunity to strategically leverage the current emphasis on autism awareness, advocacy, and research in a way that will also benefit those struggling with other developmental and mental health challenges.

Autism or autism spectrum disorder (ASD) is a group of developmental neurological conditions characterized by deficits in social communication and the presence of restricted or repetitive behaviors. ASD symptoms vary by person from mild to severe; some individuals have strong intellectual and language abilities, whereas others are cognitively impaired and may require life-long care. Many suffer from medical problems such as seizure and sleep disturbances (Doshi-Velez *et al.* 2013), as well as co-occurring mental health disorders including depression (Simonoff *et al.* 2008). Additionally, research from the USA and UK suggests that ASD typically affects the health and wellbeing of the entire family (Cidav *et al.* 2012) and poses significant long-term economic burden for society, with much of the cost attributable to long-term care and lost wages (Buescher *et al.* 2014). While ASD transcends social, cultural and geographic boundaries, research outside of high-income countries and among underserved populations is severely lacking, and the vast majority of research to date is limited to children.

Converging science suggests that ASD affects approximately 1% of the population globally (Elsabbagh *et al.* 2012). However, research methodologies and, consequently, prevalence estimates, range widely across studies. Findings from more recent research suggest that 1% may be a gross underestimate of ASD prevalence; a total-population study in South Korea found 1 in 38 children (2.64%) to have an ASD (Kim *et al.* 2011). The overall global burden of disease for ASD is significantly greater than that of fetal alcohol syndrome, attention deficit hyperactivity disorder, and intellectually disability, and the total years lost due to disability rose 30% for ASD in the 20-year period from 1990 to 2010 (Whiteford *et al.* 2013). The burden of autism is particularly acute in the developing world; poverty, malnutrition, poor education, inadequate maternal and child healthcare, and human rights violations compound challenges for both individuals and families with autism and the health, education, and social welfare systems trying to meet their needs. According to the World Health Organization (WHO) Mental Health Gap Action Program (mhGAP), where autism and other developmental disabilities are included under its child mental health priority, the development of effective public health solutions necessarily involves the negotiation of these barriers and complexities (WHO, 2008).

There is compelling evidence that early intervention for ASD can result in significant gains in language and cognitive ability, and improve long-term outcomes (Dawson *et al.* 2012), perhaps reducing lifetime cost and disability burden. However, a major barrier to improving the health and wellbeing of children and families touched by autism is the paucity of knowledge and expertise to recognize symptoms and identify ASD. The absence of effective screening in turn limits access to care and delays intervention. Without effective programs, the emergence of appropriate solutions that improve the quality of life for individuals with ASD and their families does not occur. Once individuals are identified, there is yet another bottleneck associated with the shortage of trained providers and healthcare professionals to meet their service needs.

The World Health Assembly (WHA), the governing body of the WHO represented by Ministers of Health from 194 member states, fully recognizes the challenges faced by the global autism community and appreciates the demand for feasible broad reaching and sustainable solutions. In 2014, the WHA adopted a resolution (WHA67.8) on ‘Comprehensive and Coordinated Efforts for the Management of Autism Spectrum Disorders,’ co-sponsored by more than 50 and supported by all member states. The resolution's focus on a single developmental or mental health condition was historic, but not unfounded. The challenges faced by our community are not unique to ASD, but are rather similar to those faced by the broader developmental disabilities and mental health communities. The resolution itself would not have been possible without the framework set forth in prior WHA decrees, specifically the Resolutions on disability (WHA66.9) and on the global burden of mental disorders (WHA65.4). As such, the WHA resolutions are complimentary and not contradictory, and so should be the strategies to implement them. A common thread is the recommendation for capacity building efforts that improve access to cost-effective interventions by strengthening community-based rehabilitation programs.

The WHO mhGAP, a cornerstone initiative of its Mental Health Action Plan, is designed to scale up access to mental health services through skills training of non-specialists in community care settings. Recent Cochrane (Oono *et al.* 2013) and WHO (Reichow *et al.* 2013) systematic reviews found compelling evidence that, with proper training, parents and other non-specialist caregivers may be able to effectively deliver therapies that can improve a child's social communication, language, and severity of ASD symptoms. Based on this evidence, the WHO has developed a parent skills training program for caregivers of children with developmental disorders including ASD. Although this implementation effort was specifically intended to improve the management of ASD in low- and middle-resource setting, it was also designed in the broader context of a ‘layered’ services approach, where ASD-specific services can be added-on to a more general package of care for developmental disabilities and mental health. This is one example of a strategy that, while intended to improve the lives of those affected by ASD, may also be used to improve developmental and behavioral outcomes in *all* children. Thus, it is currently being pilot tested for children with developmental disorders, including but not limited to ASD.

Country governments around the world have already committed to implementing the new WHA resolution on ASD. Countries with relatively scarce resources and with health challenges that extend well beyond ASD alone are adopting ASD-specific activities as a means for advancing the broader mental health and child development agendas. For example, in Albania, a successful program designed to train pediatricians in the basic skills needed for identifying and managing children with ASD, is now serving as the foundation for a national primary healthcare training program in mental health. The Albanian Ministry of Health is spearheading this effort, with support from local autism advocates and Autism Speaks, and framework from the WHA resolution.

The recent increase in ASD prevalence has spurred a global effort not only to raise autism awareness but also to deliver feasible, cost-effective, and sustainable solutions to individuals with autism and their families. The recently adopted WHA Resolution on ASD has helped to define a framework for developing public health policies that will enhance autism service capacity worldwide. Countries and communities should see this as an opportunity to build on the momentum that autism has created, to support the broader child mental health agenda.

Just as increased awareness of HIV/AIDS has been a key piece in advancing the global infectious disease agenda, autism awareness can be a similar catalyst for the child development and mental health agendas. The difference, however, is that unlike HIV/AIDS where treatment is specific to the retroviral pathogen; the package of psychosocial interventions that can effectively treat ASD is not specific to ASD alone. Rather, services that target social communication skills and challenging behaviors in ASD are also highly relevant to other developmental disorders and the promotion of healthy childhood development in general. Hence the reason why the ASD programs being field-tested by the WHO were developed in the context of mhGAP and are intended to address challenges across a broad range of child developmental and mental health conditions.

Similarly, the early childhood development community is taking notice, and had begun integrating child mental health and developmental delay in its priorities, in part by engaging Autism Speaks in the US Institute of Medicine forum on Investing in Young Children Globally (iYCG). The promise of transferring a more ‘generic’ child development skillset to non-specialists holds promise for improving the capacity of communities to deliver services for ASD, developmental disability, child mental health, and early childhood development alike. Such a task-shifting strategy can, in turn, improve mental health systems on the whole, creating a positive feedback-loop that reaches all sectors of the child mental health community, not just the ASD community.
